# Methylphenidate Normalizes Frontocingulate Underactivation During Error Processing in Attention-Deficit/Hyperactivity Disorder

**DOI:** 10.1016/j.biopsych.2011.04.018

**Published:** 2011-08-01

**Authors:** Katya Rubia, Rozmin Halari, Abdul-Majeed Mohammad, Eric Taylor, Michael Brammer

**Affiliations:** a Department of Child Psychiatry, Institute of Psychiatry, King's College London, London, United Kingdom; b Department of Neuroimaging, Institute of Psychiatry, King's College London, London, United Kingdom

**Keywords:** Attention-deficit/hyperactivity disorder (ADHD), error processing, methylphenidate, motor response inhibition, performance monitoring, stop task

## Abstract

**Background:**

Children with attention-deficit/hyperactivity disorder (ADHD) have deficits in performance monitoring often improved with the indirect catecholamine agonist methylphenidate (MPH). We used functional magnetic resonance imaging to investigate the effects of single-dose MPH on activation of error processing brain areas in medication-naive boys with ADHD during a stop task that elicits 50% error rates.

**Methods:**

Twelve medication-naive boys with ADHD were scanned twice, under either a single clinical dose of MPH or placebo, in a randomized, double-blind design while they performed an individually adjusted tracking stop task, designed to elicit 50% failures. Brain activation was compared within patients under either drug condition. To test for potential normalization effects of MPH, brain activation in ADHD patients under either drug condition was compared with that of 13 healthy age-matched boys.

**Results:**

During failed inhibition, boys with ADHD under placebo relative to control subjects showed reduced brain activation in performance monitoring areas of dorsomedial and left ventrolateral prefrontal cortices, thalamus, cingulate, and parietal regions. MPH, relative to placebo, upregulated activation in these brain regions within patients and normalized all activation differences between patients and control subjects. During successful inhibition, MPH normalized reduced activation observed in patients under placebo compared with control subjects in parietotemporal and cerebellar regions.

**Conclusions:**

MPH normalized brain dysfunction in medication-naive ADHD boys relative to control subjects in typical brain areas of performance monitoring, comprising left ventrolateral and dorsomedial frontal and parietal cortices. This could underlie the amelioration of MPH of attention and academic performance in ADHD.

Attention-deficit/hyperactivity disorder (ADHD) is defined by age-inappropriate inattention, impulsiveness, and hyperactivity (DSM-IV) (1). Children with ADHD have deficits in tasks of cognitive control (2,3) concomitant with reduced activation in inferior frontostriatal, cingulate, and parieto-temporal regions (4–11). Psychostimulants, such as methylphenidate (MPH), are the most effective, first-choice treatment for ADHD, improving symptoms in 70% of patients (12,13). Nevertheless, little is known on their mechanism of action. MPH is a catecholamine reuptake inhibitor with stronger dopaminergic effects subcortically and catecholamine effects in cortical regions (12,14). The behavioral and cognitive features of ADHD are thought to be mediated at least in part by a catecholamine dysfunction, with evidence for abnormal striatal dopamine transporter (DAT) levels and dopamine availability (15). It has been argued that poor inhibitory control in ADHD children may be related to poor performance monitoring, given that ADHD children, unlike control subjects, do not slow down after errors (16–18). MPH has been shown to improve inhibitory performance as well as error monitoring in ADHD children (17,19) and to upregulate abnormally low error-related evoked potentials, presumably reflecting anterior cingulate/medial frontal activity (20,21). Surprisingly, however, only two previous functional magnetic resonance imaging (fMRI) studies have investigated the acute effects of MPH on neurofunctional networks of motor response inhibition in previously medicated children with ADHD, finding reduced upregulation with MPH in defined regions of interest of caudate, anterior cingulate and frontal brain regions (22,23). Using whole-brain analyses, single doses of MPH have shown to upregulate and normalize frontal, striatal and cingulate activation in children with ADHD during divided attention (24), interference inhibition (25), sustained attention (26) and time estimation (27). No fMRI study, however, has tested for MPH effects on motor response inhibition in medication-naive children with ADHD nor on neural networks of error processing.

In this study, we investigated the effect of MPH on neural processes of error monitoring in medication-naive children with ADHD by use of a challenging tracking stop task, optimally suited to test for error detection networks because it ensures 50% of inhibition failures in every subject.

To overcome the limitations of previous ADHD fMRI studies of inhibition of regions of interest analyses and/or a medication history in patients (22,23,28), we conducted a double-blind, randomized, placebo-controlled pharmacologic fMRI experiment of the effects of a single acute clinical dose of MPH in 12 medication-naive boys with ADHD. Furthermore, we compared brain activation in ADHD patients at baseline and after MPH with that of a healthy age-matched group of control children to test for potential amelioration or normalization effects of MPH on error-related and inhibitory brain dysfunctions during the placebo condition. Given our previous findings of upregulation and normalization of frontostriatal brain activation in medication-naive children with ADHD during sustained attention, interference inhibition and time estimation (25–27), we hypothesized that MPH would upregulate and normalize typically underactivated areas of error detection in ADHD patients relative to control subjects in ventromedial and lateral prefrontal, posterior cingulate, and parietal regions (5,6,8,29). We furthermore hypothesized that MPH would normalize typical right inferior prefrontal and caudate underactivation during inhibition in ADHD compared with healthy children (5–8,29).

## Methods and Materials

### Subjects

Twelve medication-naïve, right-handed boys aged 10 to 15 years (mean age = 13, SD = 1) who met clinical diagnostic criteria for the combined (inattentive/hyperactive) subtype of ADHD (DSM-IV) were recruited through clinics. Clinical diagnosis of ADHD was established through interviews with an experienced child psychiatrist (A-MM) using the standardized Maudsley Diagnostic Interview to check for presence or absence of diagnostic criteria for any mental disorder as set out by DSM-IV (30). Exclusion criteria were lifetime comorbidity with any other psychiatric disorder, except for conduct/oppositional defiant disorder (present in one patient), as well as learning disability and specific reading disorder, neurological abnormalities, epilepsy, drug or substance abuse, and previous exposure to stimulant medication. Patients with ADHD also had to score above cutoff for hyperactive/inattentive symptoms on the Strengths and Difficulties Questionnaire for Parents (SDQ) (31). Patients were scanned twice, in a randomized, counterbalanced fashion, 1 week apart, 1 hour after either .3 mg/kg of MPH administration or placebo (vitamin C, 100 mg).

Thirteen male right-handed adolescent boys in the age range of 11 to 16 years (mean age = 13, SD = 1) were recruited through advertisements in the same geographic areas of South London to ensure similar socioeconomic status and were scanned once. They scored below cutoff for behavioral problems in the SDQ and had no history of psychiatric disorder.

All participants were above the fifth percentile on the Raven progressive matrices performance IQ (32) (IQ mean estimate controls = 100, SD = 14; ADHD = 91, SD = 9) and paid £30 for participation. Parental and child informed consent/assent and approval from the local ethical committee was obtained.

Univariate analyses of variance (ANOVAs) showed no group differences between boys with and without ADHD for age [*F*(1,25) = 2, *p* = .2] but did for IQ [*F*(1,25) = 8, *p* < .009]. IQ is associated with ADHD in the general population (33,34). We purposely did not match groups for IQ because matching ADHD and control groups for IQ would have created unrepresentative groups and therefore be misguided (35). Furthermore, IQ was significantly negatively correlated with the SDQ scores for inattention and hyperactivity (*r* = −.5, *p* < .001). We did not covary for IQ because when groups are not randomly selected, covarying for a variable that differs between groups violates the standard assumptions for analysis of covariance. When the covariate is intrinsic to the condition, it becomes meaningless to “adjust” group effects for differences in the covariate because it would alter the group effect in potentially problematic ways, leading to spurious results (35,36).

### fMRI Paradigm: Stop Task

The rapid, mixed-trial, event-related fMRI design was practiced by subjects once before scanning. The visual tracking stop task requires withholding of a motor response to a go stimulus when it is followed unpredictably by a stop signal (8,37,38). The basic task is a choice reaction time task (left and right pointing arrows: go signals) with a mean intertrial-interval of 1.8 sec (156 go trials). In 20% of trials, pseudo-randomly interspersed, the go signals are followed (about 250 ms later) by arrows pointing upwards (stop signals), and subjects have to inhibit their motor responses (40 stop trials). A tracking algorithm changes the time interval between go-signal and stop-signal onsets according to each subject's inhibitory performance to ensure that the task is equally challenging for each individual and to provide 50% successful and 50% unsuccessful inhibition trials at every moment of the task.

### fMRI Image Acquisition

Gradient-echo echoplanar magnetic resonance imaging data were acquired on a GE Signa 1.5-T Horizon LX System (General Electric, Milwaukee, Wisconsin) at the Maudsley Hospital, London. A quadrature birdcage head coil was used for radio-frequency transmission and reception. During the 6-min run of the stop task, in each of 16 noncontiguous planes parallel to the anterior–posterior commissural, 196 T_2_*-weighted magnetic resonance images depicting blood oxygen–level dependent (BOLD) contrast covering the whole brain were acquired with echo time = 40 msec, repetition time = 1.8 sec, flip angle = 90°, in-plane resolution = 3.1 mm, slice thickness = 7 mm, slice skip = .7 mm, providing complete brain coverage.

### fMRI Image Analysis

At the individual subject level, a standard general linear modeling approach was used to obtain estimates of the response size (beta) to each of the two stop task conditions (successful and unsuccessful stop trials) against an implicit baseline (go trials). Following transformation of the fMRI data for each individual into standard space and smoothing with a three-dimensional 7-mm full width at half maximum Gaussian filter, the experimental model was convolved for each condition with gamma variate functions having peak responses at 4 and 8 sec following stimulus onset to accommodate variability in BOLD response timing. By fitting these convolved model components to the time series at each voxel, beta estimates were obtained for each effect of interest. The standard errors of these beta estimates were computed nonparametrically using a bootstrap procedure designed to operate on time series data, containing serial dependencies, with repeated deterministic (experimentally determined) effects. This method is outlined in detail in a previous work (39). Two hundred bootstraps at each voxel were used to estimate parameter standard errors. Using the combined parameter estimates over all conditions, the mean fitted time series was also computed and, from the combined bootstrap parameter estimates for each bootstrap, the 95% confidence limits on the fitted time series was computed.

The second-level analysis proceeded by computing either the group differences (patients and controls) or the drug condition differences (placebo, MPH) within patients at each voxel and the standard error of this difference (using the bootstrap estimates derived earlier). The significance of these differences was then tested in three ways: 1) a simple parametric random effects (paired *t*) test, using only the group difference/placebo-MPH effect size differences; 2) a permutation test of the same random effects *t* statistic in which the null distribution was estimated by randomly swapping the signs of the differences (we used 40,000 permutations per voxel to obtain a confidence limit of .0007–.0013 for *p* value of .001); and 3) a mixed-effects test using both the effect size differences and their subject-level standard errors to accommodate first (subject) level heteroscedasticity (40). This was also conducted using 40,000 permutations per voxel.

In addition to voxelwise maps, cluster-level inference on the contrast (beta) values was performed at a family-wise error corrected threshold of *p* < .05 using the Threshold-Free Cluster Enhancement method proposed by Smith and Nichols (41). This cluster-level inference was also used for the within-group maps for each experimental condition.

## Results

### Performance

The probability of inhibition was about 50% in all subjects with no significant group differences, showing that the task algorithm worked [F(1,38) = 1; *p* < .3; Table 1].

A multivariate ANOVA between control subjects and ADHD patients under either drug condition showed a trend for a significant group effect [*F*(8,62) = 2, *p* < .09] due to a significant univariate group effect in the standard deviation to go trials [*F*(2,34) = 5, *p* < .02], which were higher in patients under either medication condition compared with control subjects (*p* < .05). Post hoc tests furthermore revealed a trend for MPH compared with placebo to slow down reaction times within ADHD patients to both go (*p* < .06) and post-error go trials (both *p* < .07) (Table 1).

## Brain Activation

### Motion

Multivariate ANOVA showed no significant group differences between control subjects and ADHD patients under either drug condition in mean or maximum rotation or translation parameters in the x, y, or z dimensions [*F*(2,38) = .9, *p* < .5].

### Within-Group Brain Activations

#### Failed Stop–Go Contrast

Control subjects activated relatively large clusters in left and right inferior frontal cortex (IFC)/anterior insula, medial frontal/anterior cingulate cortex (MFC/ACC), precentral, inferior parietal, middle and superior temporal areas, posterior thalamus and caudate, parahippocampal gyri, precuneus/posterior cingulate, occipital, and cerebellar areas.

Activation in ADHD patients under placebo was in MFC and superior temporal cortex reaching into anterior and posterior insula, caudate, and inferior parietal and occipital cortex.

Activation in ADHD patients under MPH was in a large cluster of ACC and MFC, in left and right IFC/anterior insula, left premotor cortex, right basal ganglia, bilateral middle and superior temporal, inferior parietal and occipital areas, hippocampal gyri, posterior cingulate, precuneus, and cerebellum (Figure S1 in Supplement 1).

#### Successful Stop–Go Contrast

Activation in healthy control boys was in a large cluster comprising left and right orbital and IFC, dorsolateral and MFC, insula, basal ganglia, hippocampus, posterior thalamic regions, pre- and postcentral gyri, inferior and superior parietal, temporal and occipital cortices, precuneus, and posterior cingulate.

Activation in ADHD patients under placebo was in small clusters in right MFC, supplementary motor area (SMA), right superior temporal, postcentral, left inferior and superior parietal, and occipital cortices.

Activation in ADHD patients under the MPH condition was in superior and MFC/ACC, right globus pallidus and putamen, right superior temporal and superior and inferior parietal cortex, posterior insula, and left cerebellum (Figure S1 in Supplement 1).

### ANOVA Within-Patient Comparisons in Brain Activation Between the Placebo and the MPH Conditions

#### Failed Stop–Go Contrast

**Figure 1:**
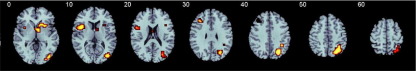
Increased brain activation with the single dose of methylphenidate compared with placebo in patients with attention-deficit/hyperactivity disorder during inhibition failures. Within-group analysis of variance in boys with attention-deficit/hyperactivity disorder comparing methylphenidate and placebo for the inhibition failure condition at family-wise error-corrected cluster-level contrast of *p* < .05. Methylphenidate compared with placebo enhanced activation in left inferior prefrontal cortex reaching into putamen, in right inferior frontal cortex/insula, reaching into caudate and putamen, in left dorsolateral prefrontal cortex, and in right inferior parietal lobe and precuneus. No brain regions were enhanced under placebo compared with methylphenidate. No within-group activation differences between drug conditions were observed for the successful inhibition contrast. The right side of the figure corresponds to the right side of the brain.

MPH contrasted with placebo elicited enhanced activation in left IFC, reaching into insula and putamen; in right IFC reaching into insula, putamen, and caudate; in left medial frontal lobe; and in left inferior parietal, precuneus, and occipital regions (Figure 1, Table 2). The placebo condition elicited no enhanced activation over MPH.

To investigate whether brain regions that differed with MPH were associated with task performance, statistical measures of the BOLD response were extracted for each subject in each ANOVA cluster and then correlated with performance variables. There was a significant positive correlation in patients between post-error reaction times and left and right IFC activation (*r* = .7, *p* < .02) and between go reaction times and right IFC activation (*r* = .6, *p* < .05) and a negative correlation between response variability and right inferior parietal activation (*r* = −.7, *p* < .02).

#### Successful Stop–Go Contrast

No significant activation differences were observed between medication conditions.

### ANOVA Between-Group Comparisons in Brain Activation Between Control Subjects and Boys with ADHD Under Either the Placebo or the MPH Conditions

#### Failed Stop–Go Contrast

**Figure 2:**
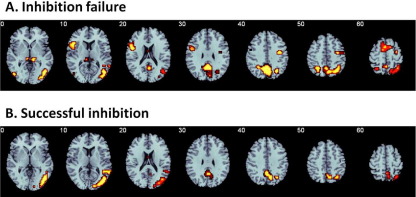
Between-group analysis of variance comparison between healthy control boys and boys with attention-deficit/hyperactivity disorder (ADHD) under the placebo condition. Significantly reduced activation in boys with ADHD under placebo compared with healthy comparison boys at family-wise error-corrected cluster-level contrast of *p* < .05 for **(A)** failed stop trials and **(B)** successful stop trials. No increased activation was observed in ADHD boys compared with healthy control boys. Under the methylphenidate condition, brain activation differences between groups were no longer observed in any of the two task conditions. The right side of the image corresponds to the right side of the brain.

Relative to control subjects, ADHD patients under the placebo condition showed underactivation in left IFC and dorsomedial prefrontal cortices (dMFC) (including pre-SMA), right premotor, superior and inferior parietal cortices, posterior cingulate/precuneus, posterior thalamus, and bilateral inferior temporo-occipital areas (Figure 2A, Table 3).

Under the MPH condition, ADHD patients did not differ from controls in any of these regions.

Post-error slowing in ADHD patients under placebo, but not in control subjects, was significantly positively correlated with activation in left IFC, premotor, dMFC, and thalamic underactivation clusters (*r* > .6 for all clusters, *p* < .05) as well as with superior parietal, occipital, and cerebellar activation (*r* < .4, *p* < .05). Standard deviation of reaction times was correlated with dMFC activation in controls (*r* = .6, *p* < .02).

#### Successful Stop–Go Contrast

Relative to control subjects, ADHD patients under the placebo condition showed underactivation in a right hemispheric network of medial temporal and inferior parietal lobes, precuneus/posterior cingulate and cerebellum (Figure 2B, Table 3). To test our hypothesis of IFC underactivation, we reanalyzed the data at a more lenient *p* value of *p* < .002 for voxelwise comparison. This elicited additional underactivation in right IFC, left and right subthalamic nuclei, and the pre-SMA (Table 3 and Figure S2 in Supplement 1).

Under the MPH condition, ADHD patients did not differ from control subjects in any of these regions.

Activation in posterior cingulate and lingual gyrus correlated significantly positively with post-error go reaction times in ADHD boys under placebo (*r* > .6, *p* < .3).

All group difference findings for both contrasts remained essentially unchanged when IQ was covaried.

### Conjunction Analysis Between Within-Group and Between-Group ANOVAs

**Figure 3:**
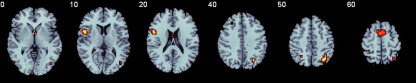
Conjunction analysis for the stop failure condition: common brain activation clusters that were significantly upregulated with methylphenidate within the attention-deficit/hyperactivity disorder group and which in addition were underactivated in attention-deficit/hyperactivity disorder patients relative to control subjects and then normalized with methylphenidate. The right side of the image corresponds to the right side of the brain.

To test whether brain regions that were upregulated with MPH relative to placebo within patients overlapped with brain regions that were reduced in patients under placebo relative to controls and then normalized with MPH, we performed a conjunction analysis by determining the voxels where the within-group ANOVA (MPH > placebo in ADHD) and the between-group ANOVA (control subjects > ADHD placebo) were both significant (42). Three clusters emerged, in left IFC (Talairach coordinates: −43, 7, 4), right SMA (Talairach coordinates: 7, 4, 59) and right inferior parietal lobe (Talairach coordinates: 32, −63, 42). Overlapping clusters are also indicated in bold in Table 3 and shown in Figure 3.

## Discussion

During error trials, ADHD boys under placebo compared with healthy control subjects showed significant underactivation in a typical error processing and performance monitoring network comprising dMFC, left IFC, thalamus, posterior cingulate/precuneus, and inferior temporoparietal regions. Among patients, MPH compared with placebo significantly upregulated activation in overlapping medial frontal, IFC, and parietal regions as well as the lenticular nucleus. Under MPH, brain activation differences between control subjects and ADHD patients were no longer observed. Reduced fronto-thalamo-parietal activation that was normalized with MPH was, furthermore, negatively associated with faster post-error reaction times in patients, which were trendwise slowed with MPH.

During successful stop trials, ADHD boys showed underactivation in a right hemispheric network of medial temporal and inferior parietal brain regions and, at a more lenient threshold, in small clusters of bilateral IFC, thalamus, and pre-SMA. Although within-patient comparison between MPH and placebo did not show significant activation differences, all underactivations in patients relative to control subjects under placebo were normalized with a single dose of MPH.

The dMFC, comprising Brodmann areas 8, 6, and 32, including pre-SMA and ACC, is a typical region of error processing and performance monitoring in adults (37,43–48) and children (29,38,49). We have previously found this region to be underactivated in children with ADHD during oddball (50) and switch tasks (4). Errors indicate violation of a reward prediction (i.e., positive performance) and have been linked to midbrain dopamine (51). Normalization with MPH of underfunctioning of this region in ADHD is in line with the notion that phasic dopamine response modulates error-related mesial frontal activation (52,53). These findings extend evidence for upregulation with acute and chronic doses of MPH in previously medicated patients with ADHD in a more rostral ACC location during tasks of cognitive control (22,28,54).

Activation in dMFC during errors triggers additional activation in functionally interconnected left IFC, as well as striatal, premotor, and parietal components of the error monitoring system, leading to post-error performance adjustments (43–45,48). IFC underactivation is one of the most consistent findings in fMRI studies in patients with ADHD, with right IFC dysfunction typically observed during inhibitory performance (7,8,11,55), in line with its role in inhibition (37,56), and left IFC during stop errors (4,29) as well as during flexible, selective, or sustained attention (4,9,26,50,57), in line with its role for performance monitoring (44,45,48,49) and saliency processing (58,59). IFC dysfunction is furthermore a disorder-specific neurofunctional deficit compared with patients with conduct (6,50,57,60) and obsessive compulsive (4) disorders. MPH thus appears to modulate an important neurofunctional biomarker of ADHD. The more predominantly left-hemispheric upregulation effect during errors may suggest a stronger effect of MPH on performance monitoring than inhibitory function in ADHD. Left IFC upregulation has previously been observed in ADHD patients in the context of an attention-demanding time discrimination task after acute (27) and 6 weeks of MPH treatment during interference inhibition (54). Structural studies have shown more normal cortical thinning in left IFC in psychostimulant-medicated compared with unmedicated ADHD children (61). Together, this raises the speculation that MPH may have a lateralized upregulating effect on left IFC structure and function.

Posterior thalamic regions have been associated both with motor response inhibition (62) and performance monitoring (48,63,64). The finding that MPH normalizes activation in this region is in line with speculation of this region's involvement in the modulation of the dopaminergic error signal (63,65,66).

The fact that lower dMFC, IFC, and thalamic activation in ADHD patients was associated with faster post-error slowing, both of which were enhanced by MPH, reinforces the role of this network for abnormal error monitoring in ADHD. Posterior cingulate and precuneus are connected with MFC and parietal areas and form part of the performance monitoring network (47,49,67,68), mediating visual spatial attention to saliency (69,70) and the integration of performance outcome with attentional modulation (48). The fact that these regions were underactivated during both inhibition and its failure is in line with a generic attention role of these areas. In line with this, we and others have previously observed underactivation in ADHD patients in these regions during inhibition errors (4,6,8,60), as well as during other salient stimuli such as oddball, novel or incongruent targets (10,26,50,71,72).

Normalization with MPH of reduced activation in typical frontoparietal regions of saliency processing and performance monitoring is consistent with the dopamine-deficiency hypothesis of ADHD given that dopamine agonists enhance stimulus salience (73). It is also in line with our previous findings of upregulation with MPH of posterior cingulate/precuneus in the same group of medication naive boys with ADHD during a target detection task, resulting in improved attention (26), and during an attention demanding time discrimination task (27). To our knowledge, normalization of inferior parietal activation with MPH has only recently been observed in ADHD patients, in the context of sustained attention (26) and interference inhibition (54).

During successful stop trials, MPH also normalized underactivation in the cerebellum, which, together with subthalamic nucleus, caudate, and IFG, forms a neurofunctional network of motor response inhibition (38). These findings extend previous evidence for cerebellum upregulation with MPH in ADHD patients during interference inhibition (54) and time estimation (27).

Within patients, MPH also enhanced activation of caudate and putamen. This is in line with previous fMRI findings of caudate upregulation in ADHD patients after acute and chronic doses of MPH during inhibition and attention tasks (22,24,54) and is likely associated with the known effect of MPH on striatal dopamine transporter blockage (14,15).

The findings of more pronounced normalization effects of MPH on abnormal performance monitoring than inhibition networks could suggest that MPH enhances generic attention and performance monitoring functions more than inhibitory capacity. This would be in line with the behavioral effect of MPH of modulating go and post-error reaction times, but not inhibition speed, which, furthermore were correlated with the reduced frontothalamic error-processing activation that was normalized with MPH. Relative to control subjects, patients only significantly differed in intrasubject response variability. Small subject numbers, a relatively older child group, and fMRI task design restrictions may have been responsible for minor behavior differences. The findings of brain dysfunctions in boys with ADHD and their normalization under the clinical dose of MPH despite minor performance differences and only trend-level improvements with MPH show that brain activation is more sensitive than performance to detect both abnormalities and pharmacologic effects. This is in line with previous findings of marked brain dysfunctions in ADHD adolescents despite no stop task impairment (7,8,50) and higher sensitivity of brain activation than behavior to show pharmacologic effects of MPH in ADHD (24,26–28,54,74).

MPH prevents the reuptake of catecholamines from the synaptic cleft by blocking dopamine and norepinephrine transporters (DAT/NET) (75,76), with higher affinity for the former (77,78). In healthy adults, MPH blocks 60% to 70% of striatal DAT in a dose-dependent manner, increasing extracellular levels of dopamine in striatum (75,79–82), as well as in frontal, thalamic, and temporal regions (83). The upregulating effects on basal ganglia, thalamic, and anterior cingulate activation were therefore likely mediated by mesolimbic striatocingulate dopaminergic pathways known to modulate error monitoring systems (63,64). In frontal regions, however, MPH upregulates noradrenaline to the same or greater extent than dopamine (84–86), via reuptake inhibition of NET that clear up both dopamine and noradrenaline (85,87–89). The upregulating effects on frontal activation, therefore, may have been mediated by enhanced catecholamine neurotransmission, in line with recent evidence that noradrenaline also plays a role in error monitoring (66,90).

A limitation of the study is that patients were tested twice, whereas control subjects were only scanned once, for ethical and financial reasons. Practice effects, however, were overcome by the counterbalanced design. Another limitation is the relatively small sample size. Minimum numbers of 15 to 20 participants have been suggested for fMRI studies (91). Repeated-measures designs, however, are statistically more powerful than independent data sets, which makes the within-subject ANOVA more robust.

To our knowledge, this is the first study to show that a single dose of MPH in ADHD upregulates and normalizes the underfunctioning of dMFC, left IFC, posterior cingulate, and parietal regions that in concert play an important role in error processing. The normalization findings of these key regions of both performance monitoring and ADHD dysfunction reinforce the association between dopaminergic neurotransmission abnormalities, ADHD, and poor performance monitoring and may underlie the behavioral effects of improving attention and school performance in boys with ADHD.

## Figures and Tables

**Table 1:** Main Variables of the Stop Task by Group

Performance Measure	Healthy Controls (*n* = 13)	ADHD Placebo (*n* = 12)	ADHD MPH (*n* = 12)
PI (in %)	49 (6)	47 (5)	52 (10)
MRT go trials (msec)	772 (118)	719 (104)	821 (154)
SD go trials (msec)	202 (52)	254 (63)	295 (103)
SSRT (msec)	205 (147)	143 (216)	166 (214)
Post-error go MRT	756 (98)	695 (123)	783 (129)

ADHD, attention-deficit/hyperactivity disorder; MPH, methylphenidate; MRT, mean reaction time to go trials; SSRT, stop signal reaction time, calculated by subtracting the mean stop signal delay (the average time between go and stop signal, at which the subject managed to inhibit to 50% of trials) from the MRT to go trials.

**Table 2:** Within-Group Analysis of Variance Differences in Brain Activation in Boys with the Attention-Deficit/Hyperactivity Disorder Between Placebo and Methylphenidate for the Inhibition Failure Condition

Brain Region	Brodmann Area	Talairach Coordinates (x,y,z)	Number of Voxels	Cluster *p* Value
Inhibition Failure Methylphenidate > Placebo				
L Inferior Frontal Cortex/Insula/Putamen/Caudate	47	−32, 15, −2	43	.001
R Inferior Prefrontal Cortex/Insula/Putamen	47	25, 14, −12	36	.0008
R Caudate		7, 4, 7	15	.0007
L Medial Frontal Gyrus	46	−32, 33, 25	11	.0005
R Inferior Parietal Lobe/Precuneus	40/7	28, 59, 42	111	.0005
R Occipital Cortex	19	25, 92, −13	27	.001
R Occipital Cortex	19	43, −78, −7	46	.0006
Placebo > Methylphenidate: No Effect				

L, left; R, right.

**Table 3:** Between-Group ANOVA Differences in Brain Activation Between Control and ADHD Boys Under Either the Placebo or the Methylphenidate Condition for the Contrast of Inhibition Failure and Successful Inhibition

Brain Region	Brodmann Area	Talairach Coordinates (x,y,z)	Number of Voxels	Cluster *p* Value
Inhibition Failure				
Controls > ADHD under Placebo				
L Inferior Frontal^a^	45/44	−47, 11, 9	78	.002
Dorsomedial Frontal Cortex/Pre-SMA^a^	6/8	0, 11, 53	74	.001
R Premotor Cortex	6	40, 4, 31	35	.002
L Thalamus (Pulvinar)		−7, −26, −2	9	.002
R Thalamus (Pulvinar)		10, 30, −7	9	.002
R Inferior Parietal Lobe^a^	40	32, 59, 42	93	.005
R Superior Parietal Lobe	7	22, 48, 59	8	.01
L Posterior Cingulate	23	−4, −48, 26	181	.005
L Precuneus	23/7	−26, −52, 42	56	.002
L Precuneus/Superior Parietal Lobe	27	−14, −44, 64	41	.005
R Inferior Temporal/Occipital Lobe	19/39	43, 63, −2	73	.002
L Occipital Gyrus	19	−43, −70, −7	8	.002
R Cerebellum		29, −85, −18	39	.004
Successful Inhibition				
Controls > ADHD Under Placebo				
R Medial Temporal Lobe	21/22	43. Forty-one, 4	40	.0004
R medial temporal lobe/occipital	39/19	47, −63, −7	104	.0005
R Lingual Gyrus	18	18, −85, 3	47	.001
R Inferior Parietal Lobe	7	32, −59, 48	27	.0007
R Precuneus/Posterior Cingulate	7/31	11, −59, 31	88	.002
Cerebellum Hemisphere		40, −59, −29	8	.003
R Inferior Frontal Gyrus/Insula^b^	47	34, 18, 0	5	.002
L Insula/Inferior Frontal^b^	44	−50, 11, 11	5	.002
L and R Anterior Cingulate^b^	32	−4, 42, 18	9	.002
L Thalamus (Pulvinar)^b^		−3, −25, 3	10	.002
R Thalamus (Pulvinar)^b^		6, −16, 3	8	.002
R pre-SMA^b^	6/8	14, 11, 53	12	.001
L pre-SMA^b^	6/8	−3, 11, 53	12	.001

*p* value for ANOVA at family-wise error-corrected cluster-level contrast of *p* < .05. No differences were observed between boys with ADHD under methylphenidate and healthy control children. ADHD, attention-deficit/hyperactivity disorder; ANOVA, analysis of variance; L, left; R, right; SMA, supplementary motor area.

a Regions that overlapped in the conjunction analysis between this ANOVA analysis and the within–patients ANOVA for areas that showed greater activation under methylphenidate than for placebo.

b Clusters only observed at a more lenient voxel–wise *p* value of *p* < .002. Boys with ADHD under placebo had no increased activation compared with control boys for either condition.
