# Supplementary material for: Methylphenidate Normalizes Frontocingulate Underactivation During Error Processing in Attention-Deficit/Hyperactivity Disorder
Source: Biol Psychiatry. 2011 Aug 1;70(3):255–62. doi: 10.1016/j.biopsych.2011.04.018 (PMC3139835; doi:10.1016/j.biopsych.2011.04.018)
Supplement: Supplement 1 [file mmc1.pdf]

Supplemental Information

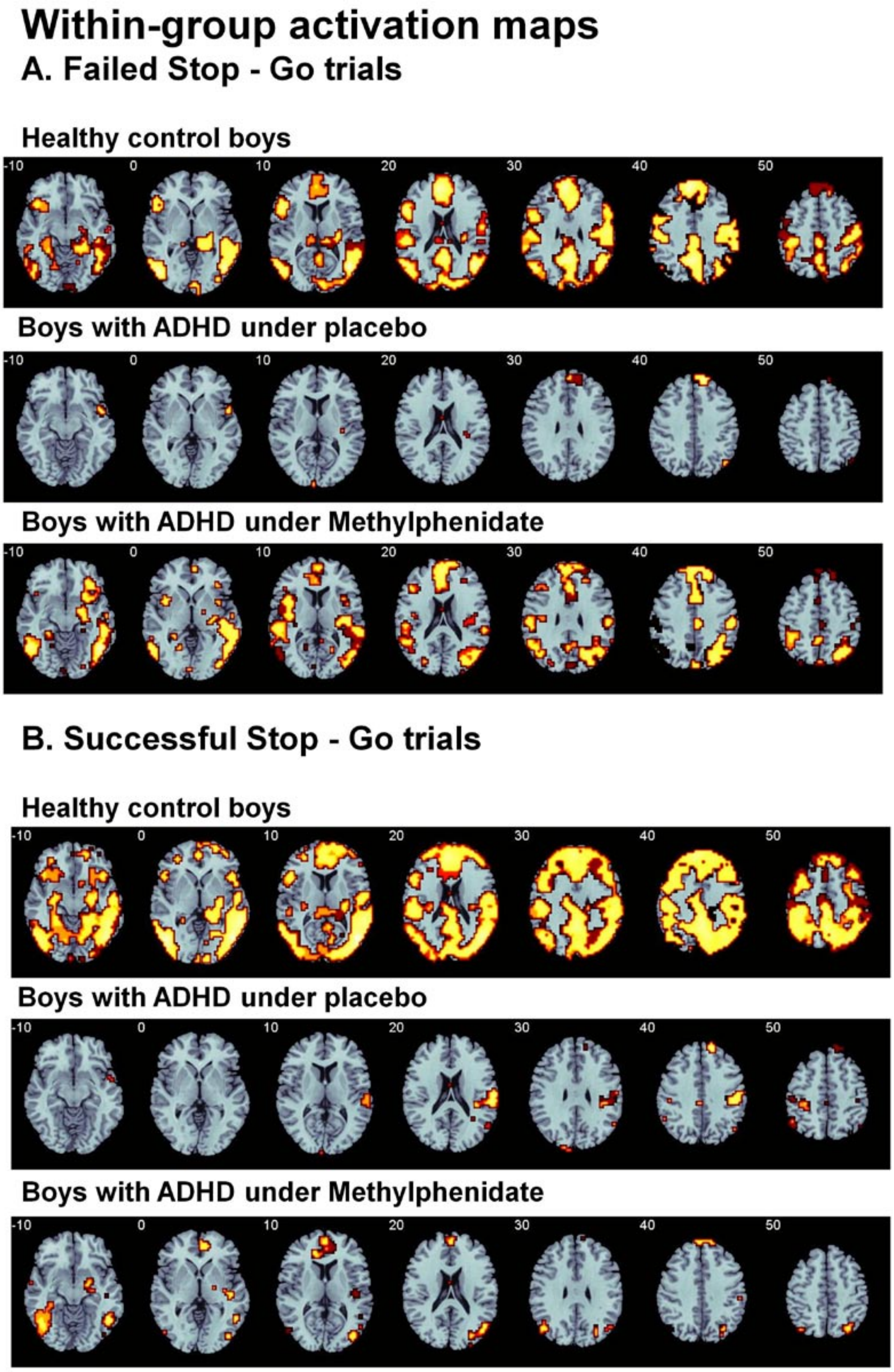

Figure S1. Axial slices for within group brain activation maps for the contrast of **(A)** Failed Inhibition and **(B)** Successful Inhibition, for 13 healthy control boys and 12 medication-naïve boys with ADHD under either the Placebo or the Methylphenidate conditions (at family-wise error-corrected cluster-level contrast of $p < 0.05$). Talairach z-coordinates are indicated for slice distance (in mm) from the intercommissural line. The right side of the image corresponds to the right side of the brain. ADHD, attention-deficit/hyperactivity disorder.

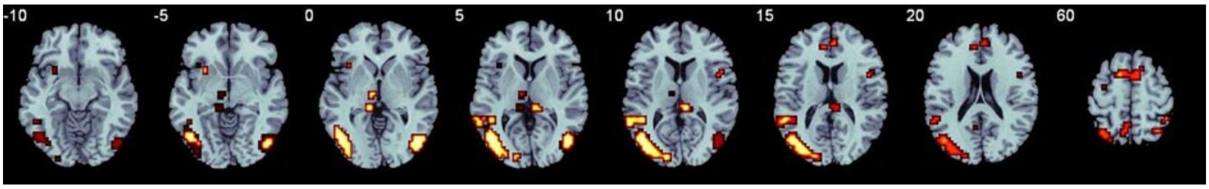

Figure S2. Significantly reduced activation in boys with ADHD under placebo compared to healthy comparison boys at voxel-level contrast of $p < 0.002$ for Successful stop-go trials. In addition to the cluster-level contrast of $p < 0.05$, additional reduction of activation was observed in right inferior frontal cortex/insula, left and right thalamus and SMA. No increased activation was observed in ADHD boys compared to healthy controls. Under the Methylphenidate condition brain activation differences between groups were no longer observed. The right side of the image corresponds to the right side of the brain. ADHD, attention-deficit/hyperactivity disorder; SMA, supplementary motor area.
